# SELF-EFFICACY PARTIALLY MEDIATES PHYSICAL FUNCTIONING AND QUALITY OF LIFE IN MIDDLE-AGED ADULTS WITH DISABILITY

**DOI:** 10.1093/geroni/igae098.2684

**Published:** 2024-12-31

**Authors:** Katie Singsank, Samantha John, Ivan Molton

**Affiliations:** University of Nevada Las Vegas, Las Vegas, Nevada, United States; University of Nevada Las Vegas, Las Vegas, Nevada, United States; University of Washington, Seattle, Washington, United States

## Abstract

Middle-age is understudied. Adults living with long-term physical disability (LTPD) face added health conditions during middle-age, heightening psychosocial stress. The role of self-efficacy in managing these conditions and their impact on quality of life (QoL) remains unclear. Regression analyses were conducted using control participants from a health promotion trial, examining whether mid-intervention self-efficacy mediated the predictive relationship between baseline physical functioning (IV) and post-intervention QoL (DV). The sample (n = 159) was predominantly female (68.6%) and aged 45-64 (M = 55.99). Regression coefficients showed statistical significance for the models examining direct effects of physical functioning on QoL, Beta =.292, t(152) = 3.759, p <.001, physical functioning on self-efficacy, Beta =.208, t(152) = 2.638, p =.009, and self-efficacy on QoL, Beta =.579, t(151) = 8.731, p <.001. A regression model was conducted examining the indirect effect of self-efficacy as a mediator between physical functioning and QoL. Physical functioning significantly predicted QoL when the mediator was accounted for in the model, Beta =.187, t(150) = 2.819, p =.005. Self-efficacy as a mediator had a stronger predictive relationship on QoL than physical functioning, Beta =.542, t(150) = 8.190, p <.001. The statistically significant indirect effects show self-efficacy partially mediated physical functioning and QoL, demonstrating physical functioning’s influence on QoL is through self-efficacy. However, its effect may lack clinical significance due to minimal Beta change. Since LTPD often limits physical improvement, interventions should prioritize self-efficacy to enhance QoL for those aging with LTPD.

